# A Systematic Review and Metanalysis on the Use of *Hermetia illucens* and *Tenebrio molitor* in Diets for Poultry

**DOI:** 10.3390/vetsci10120702

**Published:** 2023-12-12

**Authors:** Yuri Katagiri Dalmoro, Carolina H. Franceschi, Catarina Stefanello

**Affiliations:** 1 Department of Animal Science, Federal University of Santa Maria, Santa Maria 97105-900, RS, Brazil; yuri.dalmoro55@gmail.com; 2 Department of Animal Science, Federal University of Rio Grande do Sul, Porto Alegre 91540-000, RS, Brazil; carolfranceschi3@hotmail.com

**Keywords:** broiler, chitin, immune response, insect meal, insect oil, microbiota

## Abstract

**Simple Summary:**

The dietary use of insect meal is a trend that aims to reach sustainability in animal production. *Hermetia illucens* (black soldier fly) and *Tenebrio molitor* (yellow mealworm) are the most common insect species reared for larvae production since they have the capacity to transform wasted materials and food into a high-protein meal that can be included in poultry diets. However, the potential use of either *H. illucens* or *T. molitor* larvae meal and oil is not limited to the nutritional composition of their products, but it is extended to their functional effects on animal health. Recently, the functional and antimicrobial properties of dietary insect provision have also been evaluated. This manuscript provides a systematic review and a metanalysis on the use of either *H. illucens* or *T. molitor* meal and oil as ingredients or feed additives, and their effects on broilers’ growth performance and gut health. Insect meal and oil enhanced the immune status and gut microbiota, demonstrating an opportunity for the use of products that can contribute to poultry health.

**Abstract:**

Insect meal as a protein source has been considered a sustainable way to feed animals. *H. illucens* and *T. molitor* larvae meal are considered high-protein sources for poultry, also presenting considerable amounts of fatty acids, vitamins, and minerals. However, other potential components in insect meal and insect oil have been more extensively studied in recent years. Chitin, lauric acid, and antimicrobial peptides can present antimicrobial and prebiotic functions, indicating that low levels of their inclusion in insect meal can beneficially affect broilers’ health and immune responses. This systematic review was developed to study the impact of insect products on the health parameters of broilers, and a metanalysis was conducted to evaluate the effects on performance. A database was obtained based on a selection of manuscripts from January 2016 to January 2023, following the mentioned parameters. Both *H. illucens* and *T. molitor* meal or oil products had positive effects on poultry health status, especially on the ileal and cecal microbiota population, immune responses, and antimicrobial properties. The average daily gain was greater in broilers fed *T. molitor* meal compared to *H. illucens* meal (*p* = 0.002). The results suggest that low levels of insect meal are suitable for broilers, without resulting in negative effects on body weight gain and the feed conversion ratio, while the insect oil can totally replace soybean oil without negative impacts.

## 1. Introduction

Different vegetable and animal protein sources have been evaluated in diets for poultry, including dietary inclusions of insect products [1,2,3]. Most of these studies have been conducted to evaluate insect types, and also to elucidate the ideal inclusion of insect meal as an ingredient. There is a large number of insects with economic interest in animal production; however, *Hermetia illucens* (HI; black soldier fly) and *Tenebrio molitor* (TM; yellow mealworm) are the ones that can reach an industrial scale of production [4]. Meals generated from these insects contain high protein levels and sufficient quantities of fatty acids, vitamins, fiber, and minerals [5]. Additionally, *H. illucens* and *T. molitor* in either meal or oil forms are considered suitable feed ingredients for broilers as they are well accepted for poultry based on their natural behavior [6].

Feeding diets with insect products have also been considered sustainable because insects can be easily reared on a wide range of biological waste streams [6,7,8], using less land and water compared to other protein and energy ingredient sources [9]. Animal production systems based on reduced waste and pollution have become a priority, indicating that an increased efficiency of raw material utilization is needed. However, the variability in nutrient composition, the scale of production, and ingredient cost and availability are the main challenges present in the poultry chain when insect products are intended to be used.

Since more attention has been given to the precision of nutrition and animal health, aiming to improve the efficiency of poultry production, *H. illucens* and *T. molitor* larvae have been intensively studied to provide accurate information on the nutrient composition and digestibility profiles of different products for broilers. Recently, there has been an increased interest in better understanding the larvae compounds that present functional, prebiotic, or antibiotic functions and antimicrobial properties, reported to benefit broiler immunity and intestinal health [10]. In this context, chitin from the exoskeleton of the insects [11,12], the fatty acid profile with high levels of lauric acid, and the defense mechanisms provided by antimicrobial peptides [13] can also label insect meal and oil as functional ingredients for animal production. However, there is a lack of information regarding the antimicrobial properties of insect products against intestinal challenges in poultry, and there is limited information available regarding the inclusion of low amounts of insect meal, the use of insects as functional additives, and their effects on intestinal health.

Therefore, the objective of this study was to review and group the most expressive and recent literature on either *H. illucens* or *T. molitor* meal and oil sources for poultry. This study focuses on evaluating the impact of insect products on blood parameters, immune system, gut histomorphology and microbiota of broilers, based on a systematic review. This manuscript was also outlined to present the effects of insect products on broiler performance through a metanalysis.

## 2. Materials and Methods

### 2.1. Systematic Review

A literature review was performed using the PRISMA method [14] to select studies for the database construction. PubMed, Scopus, and Web of Science were used as the PICo framework electronic databases in the systematic review and metanalysis [15]. The following terms were searched: “poultry” or “broiler” or “broilers” or “chicken” or “chickens”, in combination with, “*Hermetia illucens*” or “*Tenebrio molitor*”, in addition to “chitin” or “chitosan” or “performance” or “feed conversion” or “feed conversion ratio” or “feed to gain” or “feed:gain” or “feed efficiency” or “gain:feed” or “feed intake” or “average daily gain” or “body weight gain” or “body weight” or “FCR” or “ADFI” or “ADG” or “BW” or “FI” or “health parameters” or “immune system” or “immune response” or “histomorphology” or “histology” or “morphometric” or “morphology” or “intestinal health” or “antioxidant” or “antimicrobial” or “antimicrobial peptides” or “intestinal challenge” or “challenge”.

The systematic review was conducted using published manuscripts and the cited references in the manuscripts were reviewed to identify additional studies that were relevant. All the references were exported to Mendeley software v.1.19.8 (Mendley Ltd., Elsevier, Amsterdam, The Netherlands, 2023), and each publication was independently reviewed to be selected for the next step. All the manuscripts selected for this publication were published in scientific journals from January 2016 to January 2023, considering the objective of the systematic review and the possibility of metanalysis.

For this systematic review, manuscripts mainly reporting results on the impact of HI and TM on the intestinal health of broiler chickens were selected, which basically included the effects of these products on intestinal microbiota, histomorphology and histochemistry measurements, and blood parameters. Therefore, all the main observations from the previous studies were grouped into result tables to better demonstrate the effects of dietary HI or TM products on poultry health. In the result tables, the inclusion of insect products was maintained in percentage according to each publication. Percentages of replacement were presented as inclusion levels, when possible.

### 2.2. Metanalysis

After selecting the manuscripts, a spreadsheet was created based on broiler growth performance, where columns indicate the authorship, feeding phase, ingredient inclusions, average daily feed intake (ADFI), average daily gain (ADG), and feed conversion ratio (FCR). All the data presented in the selected manuscripts were transformed to a daily basis for feed intake and body weight gain, being expressed in g/kg, whereas the FCR was presented in g:g. The obtained information for the metanalysis also includes the type of insect and physical characteristics of the ingredient (meal or oil). Additionally, manuscripts that had live or whole larvae and treatments that did not include insect products (excluding the control diet) were excluded from the metanalysis.

To perform the metanalysis, each publication received a sequential code number; this was an inter-code with a sequential code plus a number that represented the treatment. The codes were repeated to indicate different phases of the same treatment (for example, manuscript 10 and treatment 01 for the starter phase = 1001; manuscript 10 and treatment 01 for grower phase = 1001; manuscript 10 and treatment 02 for the starter phase = 1002…). This sequential code allowed us to study the effects of treatments in different growing phases, analyzing values and finding evaluated means. The feeding phases were used for categorical analysis, as follows: starter (1 to 21 days), grower (21 to 35 days), and finisher (35 to 42 days). Afterwards, the spreadsheet was set and the ADG, ADFI, and FCR variables were calculated as a percentage of the respective control in two ways. First, the response for the given performance parameter with the control treatment in each publication, separated by feeding phase, and the control diet were considered 0 (zero). This information was presented as the variation between the control and treatments in percentage. Secondly, the greatest results for the ADG and ADFI of each phase were considered 100%; however, for the FCR, the lowest (best or improved) result was considered 100% for analyses. Results were separated into the control treatment or treatments with either HI or TM insect products at low inclusion levels (≤100 g/kg for meal and ≤25 g/kg for oil) and high inclusion levels (>100 g/kg for meal and >25 g/kg for oil). This procedure was used to reduce the variability among studies in the database [16].

In the present manuscript, the metanalysis was performed to combine results from several assays in the database to compare the effects of the two insect products (HI and TM meal or oil) on broiler performance. Graphical analyses are presented to show the results of each manuscript and to obtain an overview of the data heterogeneity. Still, this procedure was divided into the type of insect products, meal or oil forms, and feeding phases that were included in the same graphs for better visualization. For the metanalysis, at the end, the 25 selected publications containing information regarding the ADFI, ADG, and FCR were used to create 8 graphs with an exploratory analysis, demonstrating the effects of insects as ingredients on broiler performance. Also, the variation in the ADFI, ADG, and FCR of broilers fed diets with either insect meal or oil, as well as an analysis of variance to compare both insects, were expressed in two tables.

### 2.3. Statistical Analysis

Data were submitted for an analysis of variance to show the effects of the inclusion levels of the insect products on growth performance. This was performed using the univariate procedure and the Shapiro–Wilk test of Minitab^®^ v.21.4 Statistical Software. After, the performance responses were analyzed for variance–covariance. The model included the code of the manuscript as a random effect. Insect meal and oil were fixed effects. Mean differences were considered significant at *p* ≤ 0.05 using the Tukey test.

## 3. Results

**Figure 1:**
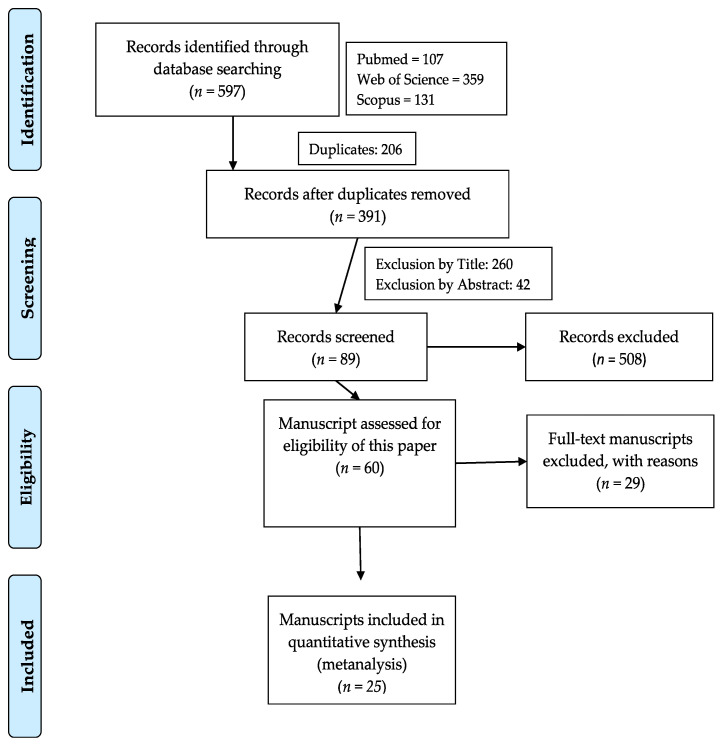
Flow diagram of the systematic review process.

The literature review resulted in 597 manuscripts that were identified and imported from the database, as shown on the PRISMA flow (Figure 1). In the identification, a total of 508 studies were excluded due to an inconsistent title, abstract, or duplicates. For the present publication, a total of 60 manuscripts were eligible, presenting growth performance information and health impacts in broiler chickens. After full evaluation, 29 publications were removed from the metanalysis due to a lack of information on performance data; these precluded the correction for ADFI, ADG, and FCR or were removed due to missing information regarding a description of the diets. A total of 25 studies were retained, as described in Figure 1 and Table 1. The nutritional and aminoacidic composition of either the HI or TM meal are presented in Figures S1–S4, and 16 studies that used insects as ingredients in the diets and also presented the nutrient composition of the meals were obtained. Data were corrected to a dry matter basis when needed.

### 3.1. Systematic Review

The results of the systematic review of broilers fed diets with HI and the impact on their health parameters are shown in Table 2. This table presents the authorship, overall information about the diet and insects, inclusion levels, and the main observed effects. The publications evaluated HI meal or oil separately, with variable inclusion levels of these products. Few studies replaced soybean meal (SBM) with HI meal or soybean oil with HI oil in different percentages of replacement. Regarding the main effects, previous publications mainly evaluated the microbiota, immune responses, intestinal histomorphology, and blood parameters of the broilers. The results of the histomorphology and blood parameters were variable, ranging from no effects to improvements in villus height and blood triglycerides, monocytes, and red blood cell distribution. Some publications demonstrated that there is a potential to use HI meal or oil to improve the relative abundance of beneficial microorganisms and to decrease *Clostridium* in the gastrointestinal tract (GIT).

Table 3 presents the results of the systematic review of broilers fed diets with *T. molitor* and the impact on health parameters. The authorship, overall information of diets and insects, inclusion levels, and the main observed effects are shown. Almost all the publications evaluated TM meal and few studies tested TM oil in broiler diets. Also, the majority of the studies were designed to evaluate different inclusions of TM meal in diets for broilers as a protein source, and few studies tested the supplementation of full-fat TM meal as a feed additive. The relative abundance of microorganisms was the main studied effect in the previous publications, followed by measurements of the immune responses and blood parameters. Interestingly, some publications reported that TM meal inclusion in broiler diets was effective in decreasing populations of *Escherichia coli*, *Clostridium*, *Coprococcus*, and *Ruminococcus* in different trials.

Table 4 shows the effects of *H. illucens* and *T. molitor* in broiler diets and their impact on broiler health characteristics based on a systematic review. There are fewer studies evaluating both HI and TM for broiler diets in the same experiment compared to experiments evaluating only one insect species. Two studies were developed to evaluate the microbiota with different provisions of HI or TM products, and one publication supplemented HI or TM. Until the date of this systematic review, there were no publications found to evaluate both HI and TM in the same diet.

### 3.2. Metanalysis

**Figure 2:**
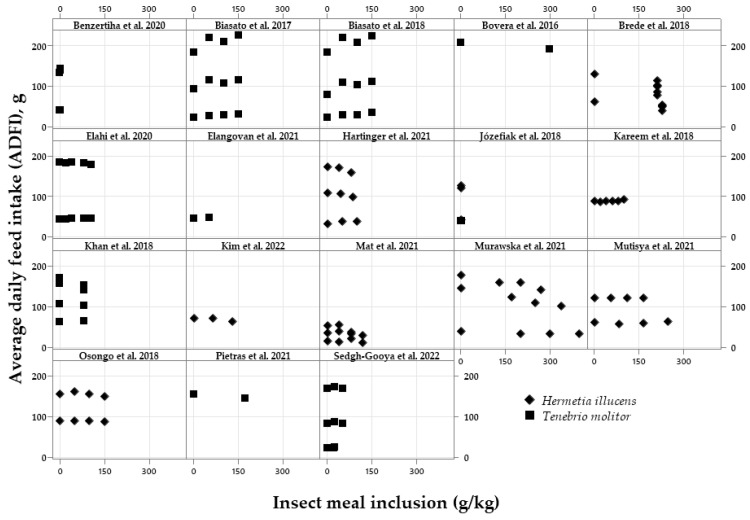
Exploratory graphs with the effects of increasing dietary inclusions of *Hermetia illucens* or *Tenebrio molitor* larvae meal on the average daily feed intake (ADFI) of broilers [17,18,19,20,21,23,24,27,28,29,31,32,33,34,35,36,37,40].

**Figure 3:**
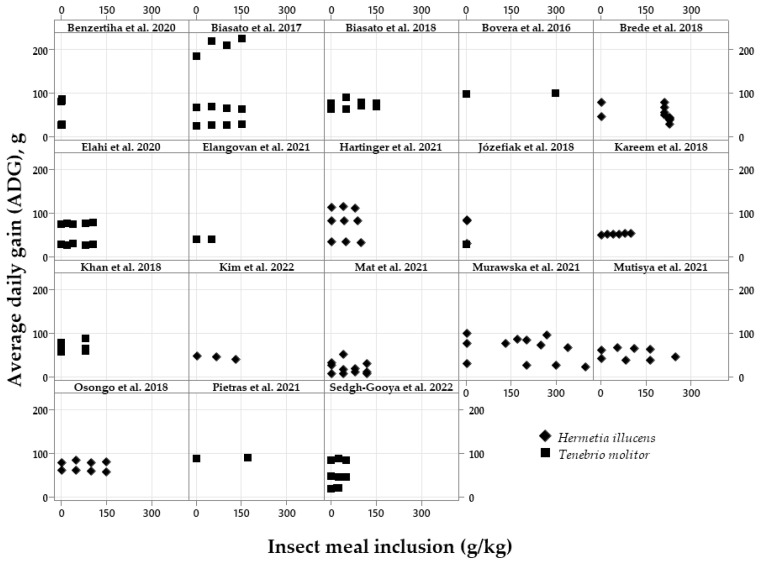
Exploratory graphs with the effects of increasing dietary inclusions of *Hermetia illucens* or *Tenebrio molitor* larvae meal on the average daily gain (ADG) of broilers [17,18,19,20,21,23,24,27,28,29,31,32,33,34,35,36,37,40].

**Figure 4:**
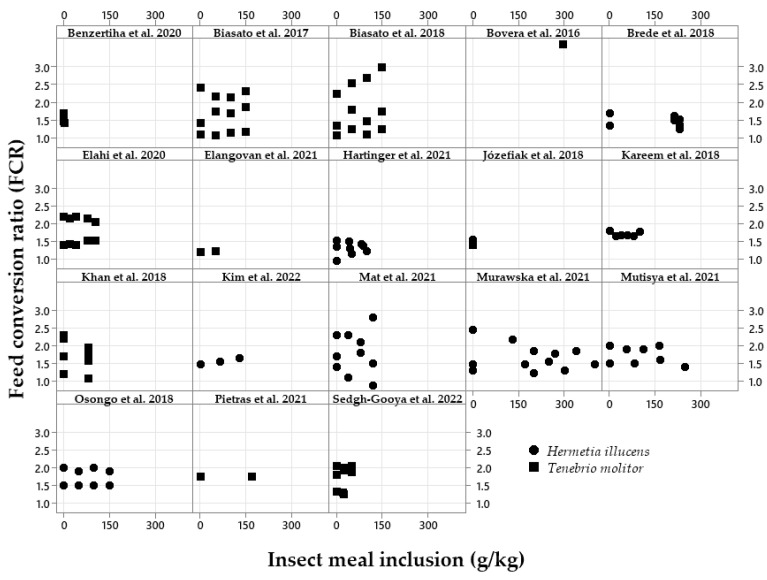
Exploratory graphs with the effects of increasing dietary inclusions of *Hermetia illucens* or *Tenebrio molitor* larvae meal on the feed conversion ratio (FCR) of broilers [17,18,19,20,21,23,24,27,28,29,31,32,33,34,35,36,37,40].

**Figure 5:**
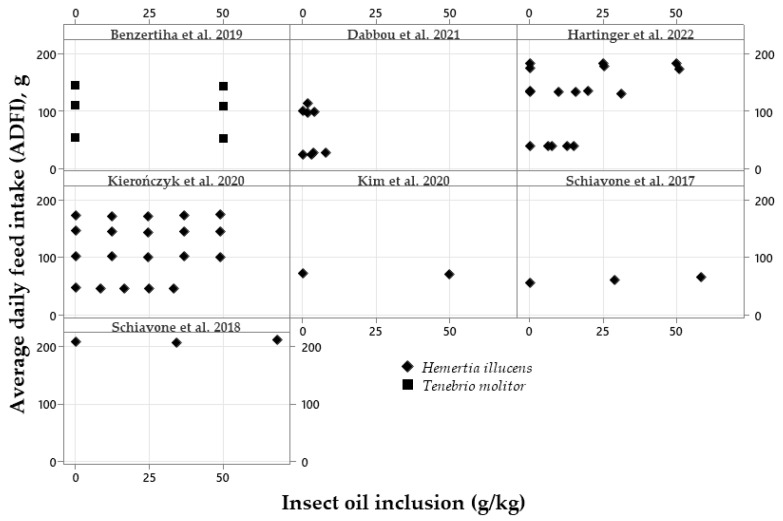
Exploratory graphs with the effects of increasing dietary inclusions of *Hermetia illucens* or *Tenebrio molitor* oil on the average daily feed intake (ADFI) of broilers [6,20,24,26,30,38,39].

**Figure 6:**
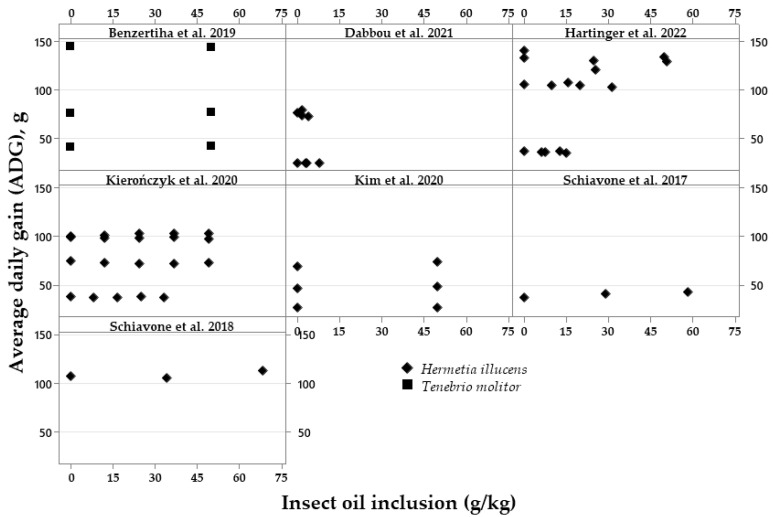
Exploratory graphs with the effects of increasing dietary inclusions of *Hermetia illucens* or *Tenebrio molitor* oil on the average daily gain (ADG) of broilers [6,20,24,26,30,38,39].

**Figure 7:**
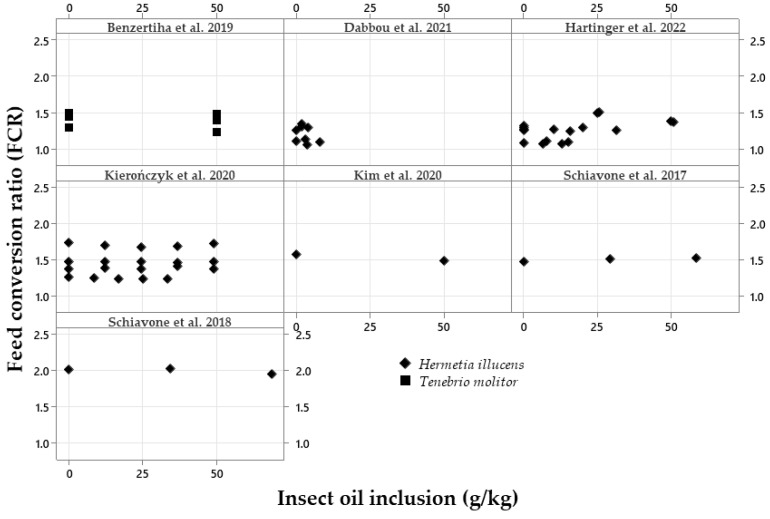
Exploratory graphs with the effects of increasing dietary inclusions of *Hermetia illucens* or *Tenebrio molitor* oil on the feed conversion ratio (FCR) of broilers [6,20,24,26,30,38,39].

The exploratory graphical analyses are shown in Figure 2, Figure 3, Figure 4, Figure 5, Figure 6 and Figure 7. The ADFI, ADG, and FCR of broilers fed HI or TM larvae meal are presented in Figure 2, Figure 3 and Figure 4, respectively. The figures allowed us to observe the biological coherence between HI or TM larvae meal inclusion and the growth performance parameters of broilers. Also, with the metanalysis, it was possible to obtain a general view of the data consistency and heterogeneity, as well as to observe the data distribution regarding dietary insect levels. Since the feeding phases are within the same graphs in the present manuscript, we can observe the results of different phases, observing points that are closer or farther to the Y axis in the graph of each publication.

Figure 2 shows the ADFI from 0 to 200 g, with the HI or TM larvae meal inclusion ranging from 0 to 300 g/kg. A decrease in the ADFI can be seen with the increasing levels of HI meal in three manuscripts [19,22,24]. On the other hand, increasing inclusions of TM meal do not seem to change the ADFI.

The effects of insect meal inclusions on broiler ADG are shown in Figure 3. For this parameter, the ADG does not seem to change with increasing levels for both insects, but there is a publication [35] demonstrating an increase in the ADG in the finisher phase.

The FCRs of broilers according to the insect meal inclusion are presented in Figure 4. This shows that no changes in the FCR between the insect sources were observed. The FCR results moved from an increased FCR using increasing levels of TM meal [34,35] to improvements in the FCR [31]. With HI meal inclusions, an increase in the FCR was also reported [25,51].

The effects of increasing levels of HI or TM oil on broiler performance are described in Figure 5, Figure 6 and Figure 7. Figure 5 shows the ADFI from 0 to 200 g, when broilers were fed HI or TM larvae oil ranging from 0 to 50 g/kg. Increasing levels of insect oil did not affect the ADFI of broiler chickens. One publication was found to evaluate TM oil for broilers in floor pens [55] and another did so in metabolic cages [61].

In a similar manner to the ADFI results, the ADG of broilers was not affected by increasing levels of either HI or TM oil (Figure 6).

Figure 7 shows the FCR from 1.0 to 2.5 g:g, with HI or TM oil ranging from 0 to 50 g/kg. The insect oils, independent of the source, did not seem to have any effect on this parameter.

Since the feeding phases of each publication were included in the already presented graphs, the high heterogeneity of the results was implied. The ADFI tended to decrease with the inclusion of insect meal in most of the manuscripts, while the ADG or FCR were stable throughout the inclusion of insect sources. Using insect oil, the majority of the broiler performance results were similar among inclusions.

**Figure 8:**
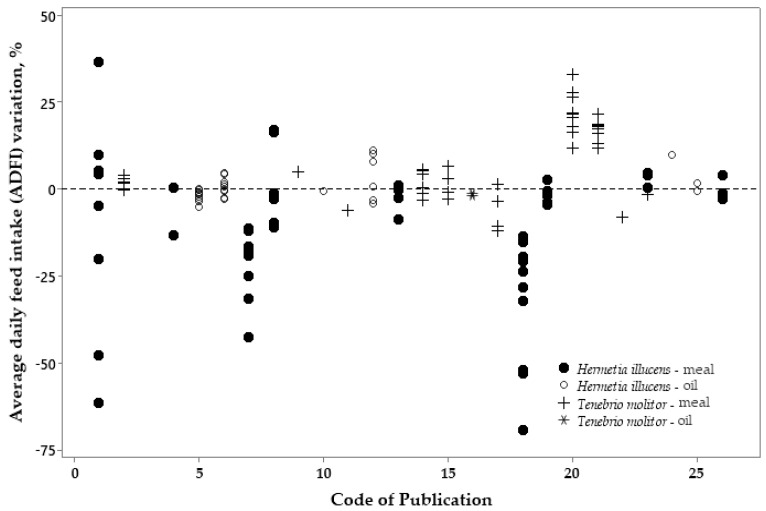
Variation (%) in the average daily feed intake (ADFI) responses of broilers fed the control diets (zero in the Y axis) or diets with *Hermetia illucens* or *Tenebrio molitor* sources.

**Figure 9:**
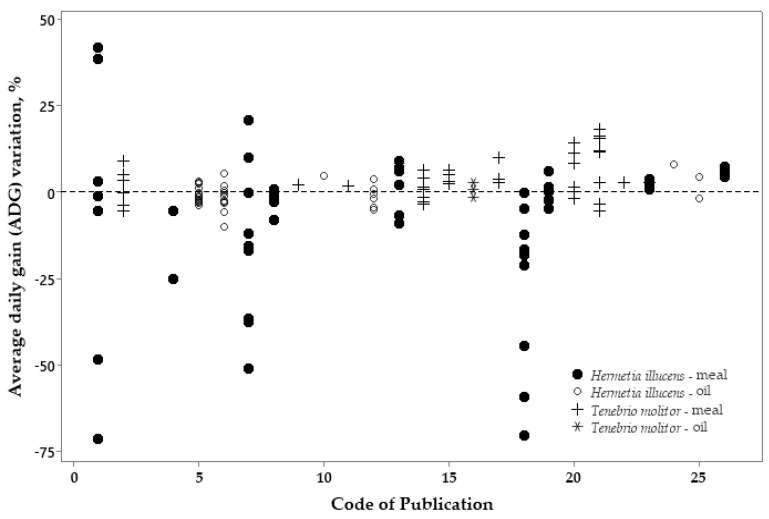
Variation (%) in the average daily gain (ADG) responses between broilers fed the control diets (zero in the Y axis) or diets with *Hermetia illucens* or *Tenebrio molitor* sources.

**Figure 10:**
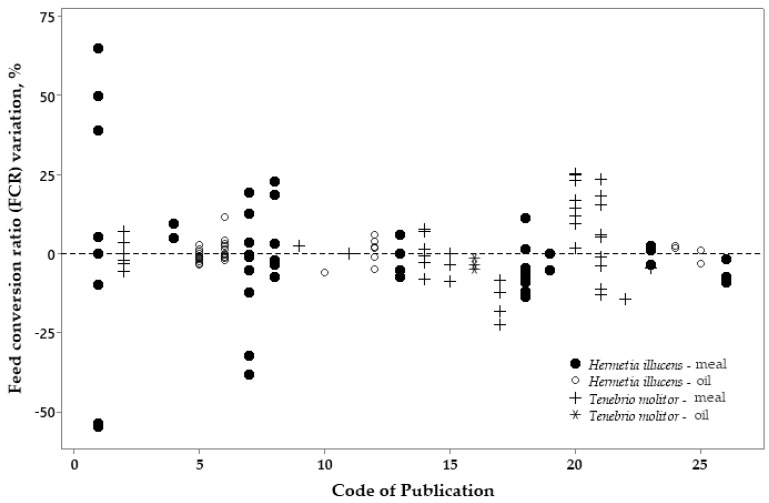
Variation (%) in the feed conversion ratio (FCR) responses between broilers fed the control diets (zero in the Y axis) or diets with *Hermetia illucens* or *Tenebrio molitor* sources.

The dispersion graphs below (Figure 8, Figure 9 and Figure 10) show the percentage of variation in the ADFI, ADG, and FCR of broilers fed either HI or TM meal and oil related to the control diets (which is zero in the Y axis) using all selected publications.

Figure 8 presents the variation in the ADFI between treatments with the inclusion of insect products and the control diet for broilers. The variation between the control and treatment diets with HI larvae meal is high and negative. For HI oil, TM meal, and TM oil, the ADFI did not vary from the insect inclusion to the control diet in most of the publications.

The ADG variation between the control diets and diets with insect meal sources (Figure 9) indicates high variation. Negative values seem to be more present when TM meal was used. There was no observed variation between the use of insect oil or TM meal compared to the control diets.

Figure 10 shows the variation in the FCR of broilers fed diets containing different levels of insect meal or oil compared to the control diets. This parameter followed the ADFI and ADG results, with the most negative variation values found for the HI larvae meal and a higher variation found for the TM meal. The oil sources did not affect the variation in the FCR.

The variance–covariance analysis comparing both insect sources is presented in Table 5 and Table 6. Table 5 shows the variation in the percentage of HI and TM inclusion against the control diets and a comparison between the two insects. Significant differences in the variance for the performance results were observed in the ADFI, where there was a difference of −10.70 in the ADFI using HI meal compared to TM meal, which had +7.20 variance in the meal form. The ADG followed the same premises, as −7.92 was obtained for HI meal and +4.05 for TM meal. For oil sources, there were differences (*p* ≤ 0.05) in the ADFI and FCR. The variance in the ADFI was +0.60 for HI oil and −4.25 for TM oil, while +0.41 was found in the FCR for HI oil and −3.54 for TM oil.

Table 6 shows the effects of low or high inclusions of either insect meal or oil, without separating the insect species. For the insect meal, the high inclusion of insect meal decreased the variation in the ADFI and ADG of broilers (*p* < 0.001), without effects on the FCR. For the insect oil inclusion, no differences were found.

## 4. Discussion

### 4.1. Impacts of Dietary Inclusions of Insect Products on Poultry Health

Insect-based products are receiving great attention as potential ingredient sources for animals. However, it is difficult to obtain an evidence-based view from the large volume and diversity of information that has been increasingly generated. For this reason, a systematic review with a metanalysis can be a useful method to summarize the findings of all the previous studies. Thus, this manuscript contains all searched studies that explored insect products, either in meal or oil forms, used in diets for broilers and their effects on growth performance and health parameters.

Insect meals have been evaluated and utilized in broiler diets mainly as dietary protein sources and as functional feed additives. Most of the publications tested the ability of inclusions of *H. illucens* (black soldier fly) and *T. molitor* (yellow mealworm) to partially replace soybean meal, whereas the studies evaluating insect oil were often designed to replace soybean oil. However, feeding insect products to broilers still generates variable results due to the variability in their composition, the different tested inclusion levels and production systems, as well as the lack of information on the digestibility and functional properties of insect products.

Commercial poultry production demands high amounts of ingredients; however, the practical and economical approach of replacing soybean meal with insect meal is still a challenge. For this reason, the benefits of low inclusions of insect products have been recently addressed. Therefore, more data are needed to better understand whether insect meal or oil sources can beneficially impact the intestinal health, immune system, and antimicrobial functions of broilers. Considering these premises, it is also important to evaluate the effects of insect products on the health status of broilers reared under enteric challenge conditions, which is something that has not already been published in the literature. The only published material found through the systematic review evaluated broilers fed HI meal and reared under a *Salmonella enteritidis* Gallinarum challenge [49].

As an avian species, broiler chickens search for and ingest insects as a natural behavior. The utilization of scattered larva has enhanced this natural behavior, affecting the leg health and welfare of chickens [41,48]. Since poultry have evolved to have this behavior, it has been highlighted that acid chitinases are present in the glandular stomach [62]. Chitin is one important component that constitutes the exoskeleton of insects and it has been associated with a decrease in nutrient digestibility when present at high levels in the diets of poultry [57]. However, chitin is also known as a natural antimicrobial component [63,64], and besides chitin, the presence of different antimicrobial peptides identified in both *H. illucens* and *T. molitor* [65] can contribute to poultry health. For this reason, the chitin composition in different insect meal products and the possible beneficial properties of chitin and antimicrobial peptides on poultry health should be studied more in the future.

The evaluation of poultry health uses variable and different approaches, and the present review was developed to better understand the effects of the main insect meals and insect oils available for poultry diets. The present systematic review intended to demonstrate the effects of insect products whose effects on broiler microbiota, gut histomorphology, and blood parameters had already been reported. The importance of poultry microbiota in gut health is related to its capability to ferment carbohydrates and dispose energy and fatty acids as substrates for the GIT [66]. The most predominant genera of microorganisms in the GIT of broilers are *Clostridium*, *Ruminococcus*, *Lactobacillus*, and *Bacteroides* [67], which have a different relative abundance in each intestine segment [68]. In the current study, it was observed that the population of beneficial bacteria is affected by the dietary inclusion of either insect meal or oil for broilers. The presence of lauric acid in insect meal could be one factor that influences bacteria population in the GIT since this fatty acid affects the proliferation of potentially pathogenic bacteria. Kierończyk et al. [43] reported that increasing levels of butyric and lactic bacteria were observed using the insect oil, which also decreased the crop pH and helped to control the pathogenic bacteria that usually do not tolerate an acidic pH. However, the authors observed an increased proliferation of the *Bacteroides–Prevotella* cluster, *Enterobacteriaceae* and *C. perfringens*, probably due to the feed retention that was correlated to the antimicrobial effect and the availability of lauric acid in the crop.

The *Ruminococcaceae* and *Lachnospiraceae* families have been reported to produce butyric acid [69], which is used as an energy source for enterocytes in the gut [70]. Chitin from insects can affect the production of butyric acid [56], and a previous study indicated that low levels of insect full-fat meal (0.2%) reduced pathogens such as *C. perfringens* [30]. On the other hand, high doses of HI meal improved the volatile fatty acid profile of the caeca content of broilers [19]; however, the authors only described the correlation between the fatty acid profile of the HI meal in the caeca, without relating chitin to the prebiotic effects. The prebiotic functions provided by chitin from insects were previously described as favorable and able to improve gut health [71]. The mucin dynamics, when using insect meal, were suggested to beneficially improve the histological parameters correlated with microbiota due to the production of volatile fatty acids [47].

Protein and amino acids that escape from digestion and absorption in the hindgut can be fermented in the caeca being used by bacteria [68]. This can extend the nutritional functions of feeding protein sources once they can be used by the microbiota, impacting the gut health of animals. The caeca microbiome, as affected by *H. illucens* and *T. molitor* meals, has already been described [52,54]. Indirect approaches using metabolites can be used, as demonstrated by Hartinger et al. [20] and through a principal component analyses [53]. In the present study, it was observed that insect meal affected the gut microbiota, especially Firmicutes, *Lactobacilli*, and Bacteroides populations. The positive impacts of insect products on the microbiota were indicated, since lower rates of Bacteroidetes and higher rates of *Lactobacilli* are correlated with good gut health [47,53]. The *Ruminococcaceae* family is also important for intestinal health and it was affected by insect meal [47]. Based on the results, HI and TM probably have different paths in microbiota modulation and their effects differed from each other [37,60]. Also, the region of the GIT in which the microbiota populations were evaluated is variable among the peer-reviewed publications, possibly influencing the results.

In the present study, we observed that the histomorphology findings were not consistent with the inclusion of insect meal or oil in diets for broilers. Some publications that enhanced this affirmation replaced soybean oil with 50% to 100% HI oil and did not observe alterations in gut histomorphology [6,32]. However, there are reports of replacing soybean oil with 50% insect oil where an increased villus height in the ileum was observed [46], while similar results for the jejunum villus height were reported using 50% and 100% oil replacement [20]. One publication [34] indicated that a 15% inclusion of TM meal decreased the villus, deepened crypts, and decreased the villus/crypt ratio compared to 5 and 10% for broilers, whereas Biasato et al. [35], evaluating the same TM meal, did not observe any differences in the histomorphology parameters.

Higher monocytes and hemoglobin were reported in broilers fed HI meal [17], replacing fish meal. Serum lipids and cholesterol were investigated using HI meal, and a decreased cholesterol was found in blood samples. This was associated with the presence of chitin [19], indicating that the chitin could decrease lipid absorption by binding to lipids; as a result, the plasma cholesterol decreased. Using TM meal for broilers increased the albumin/globulin ratio and serum globulin levels [51]. These parameters were also related to the chitin levels, which could work as a prebiotic and boost the immune response. Increased erythrocytes and decreased albumin and gamma glutamyl transferase (GGT) using TM meal were reported by Biasato et al. [35]. These findings were seen as good results, since high GGT levels indicate liver and bile flow disorders.

The inclusion of HI oil as a functional ingredient for poultry diets has been increased because lauric acid presents antimicrobial properties [6,43]. The impacts of the 100% replacement of soybean oil with insect oil were not negative according to our systematic review. Low levels of cholesterol in blood samples demand low bile synthesis, indicating that these animals have highly efficient fat utilization. Additionally, this result, together with low levels of alanine aminotransferase, can be related to good liver health. Serum triglycerides reduced when TM oil was compared to poultry fat [55]. The authors also indicated that the high levels of polyunsaturated fatty acid in TM oil could reduce the conversion of acetyl-Coa to triglycerides in the liver, affecting blood triglycerides in broilers.

The use of insect meal has been demonstrated to potentially improve the immunity status in poultry, crustaceans, and fish [72,73,74]. Even though publications with other animal species were not selected in the systematic review, pigs fed HI meal had decreased diarrhea at weaning age and improved intestine homeostasis under the presence of *Escherichia coli*, which promoted levels of mucin-1 and mucin-2 while regulating the expression of antimicrobial peptides through the TLR2-NF-κB/MAKP signaling pathway [75]. Also, the authors observed decreased pro-inflammatory and increased anti-inflammatory cytokines at finishing ages using HI meal in diets [76]. As for dogs and cats, there are reports indicating improvements in the antioxidant status [77], with reduced TNF-α and increased concentrations of superoxide dismutase and glutathione peroxidase in the serum of dogs fed diets with HI meal [78]. However, more studies are needed to better understand the effects on anti-inflammatory and immunity parameters [79] in companion animals but also in poultry. For the immunology effects in broilers, a decrease in white cells and an improved performance were shown [45]. Also, under a viral challenge, there was a reduction in the load of avian infectious bronchitis virus (IBV) in the trachea and kidney using 10% HI meal [44], and an increase in the survival rate of broilers, with less damage to the trachea tissues. The authors summarized that interferon-gamma (IFN-γ) was activated in mRNA levels and that this was involved in the activation of T-cells. Still, the interleukin-2 levels increased and promoted CD8+ lymphocyte responses. Finally, the major histocompatibility complex-I (MHC-I) molecules that control CD8+ increased, and with the promotion of CD8+ lymphocytes, the elimination of avian IBV increased when the diets included HI meal.

An experimental challenge with *Salmonella enteritidis* Gallinarum was conducted in broiler chickens fed HI meal [49]. The authors observed an increase in the birds’ survival rate followed by a decrease in Gram-negative colonies in the liver, spleen, bursa of Fabricius, and caeca. However, the mechanism of HI meal against S. *enteritidis* Gallinarum was not clear. Evaluating a fermented probiotic from TM meal for broilers under *Salmonella* and *Escherichia coli* challenge, Islam and Yang [58] reported decreased mortality and improved levels of immunoglobulin (Ig) IgA and IgG. This study showed a reduction in the caeca *E. coli* and *Salmonella* counts, indicating that the combination of the antimicrobial effects of chitin and antimicrobial peptides from larvae could contribute to a reduction in pathogenic bacteria. The results highlight the possibility of evaluating the effects of insect products at low levels on pathogenic bacteria when broilers are reared under intestinal challenges.

### 4.2. Metanalysis: Exploratory Analyses of Insect Products on Broiler Performance

Regarding the growth performance of broilers fed diets with the inclusion of insect products, a metanalysis was previously published [80], but did not discriminate the insect products. During the writing of this manuscript, another metanalysis was published to predict the metabolizable energy of HI meal and its effects on performance [81]. In the current manuscript, the metanalysis was conducted to compare the results of performance parameters such as the ADFI, ADG, and FCR between the insect sources.

The database utilized for the present metanalysis consisted of manuscripts published in scientific journals between January 2016 and January 2023, as presented in Table 1. Different sources of protein as main ingredients, variable broiler ages and genetics, and different insect products were reported, and this could be a reason for the heterogeneity of the results reported in Figure 2, Figure 3, Figure 4, Figure 5, Figure 6 and Figure 7. Regardless, Figure 8, Figure 9 and Figure 10 indicate a greater growth performance in broilers fed TM meal compared to HI meal, and this was confirmed in Table 5 for the ADFI (*p* < 0.001) and ADG (*p* = 0.002). A different outcome was observed for the oil sources, probably due to the lower number of available publications with TM oil compared to TM meal and HI oil. During the construction of this database, no manuscripts were found evaluating, in the same study, the effect of both insects as ingredients on broiler performance. For this reason, further experiments can be performed using different insect sources in the same diet since the availability of insect products can be variable worldwide.

Most of the publications regarding the inclusion of insect products for broilers have been conducted using insect meal to replace soybean meal. The levels of inclusion or replacement of HI or TM meal usually ranged from 5 to 15%. However, this can be not cost-effective for commercial broiler production and, for this reason, evaluating the lower inclusion of insect products can be highlighted. In the present study, the inclusion of insect meal at low levels had a similar effect on growth performance to the control treatment in broilers without any intestinal challenge. No negative effects on performance were reported when evaluating low levels of insect meal for broilers [18,21,22,34]. Dietary inclusions of insect meal with levels higher than 1% and lower than 10% should be studied more since this can result in different effects compared to very low or high levels. This evaluation can improve the information on the effects of low inclusions of insect meal on broiler nutrition and health, also allowing for more cost-effective results. For the insect oil, no differences were found with low or high inclusion levels, and no negative effects on broiler performance were presented [38]. No data on full soybean oil replacement with TM meal were found in the literature.

In conclusion, the use of insect products in poultry diets affected broilers’ health, demonstrating positive effects on the modulation of microbiota. This can be noticed through the different effects of various phylum, classes, families, and genera of bacteria reported in this review, which are mostly from caeca content. The reduction in pathogenic bacteria and the enhanced production of short-chain fatty acids are also characteristics of insect inclusions in poultry diets. Nevertheless, the immunology system is indeed improved, and reduced mortality was observed in challenged animals, with positive impacts on immunoglobulins, interleukins, and lymphocytes. To better understand the microbiota mechanism, future studies should be conducted on the inclusion levels of insect products and the different intestinal challenges that can be found in production systems. For the performance data, *T. molitor* had better results if compared to *H. illucens*; also, a high meal inclusion (>100 g/kg) of insects decreased broiler performance, as already reported in some manuscripts. The dietary utilization of insect oil did not affect any performance parameters and it seems that *H. illucens* oil can be used to completely replace soybean oil, whereas more studies are needed using *T. molitor* oil. Also, more data are needed to evaluate the effects of insect oil on poultry health when aiming to provide more robust conclusions in the presence of an enteric challenge.

## Figures and Tables

**Table 1:** Summary of live production studies used in the metanalysis.

Reference	Code of Publication	Insect Used ^1^	Feeding Phases ^2^	Genetic/Sex ^3^	Protein/Oil Source ^4^	*n* ^5^
Mat et al. [17]	1	HI	Str, Grw, Fin	Cobb/male	FM, SBM	360
Sedgh-Gooya et al. [18]	2	TM	Str, Fin	Arbor Acres	FM, SBM	180
Kim et al. [19]	3	HI	Str, Grw	Ross/male	SBM, CGM	126
Kierończyk et al. [6]	4	HI oil	Str, Grw	Ross	SBM	960
Hartinger et al. [20]	5	HI	Str, Grw	Ross/male	SBM	432
Murawska et al. [21]	6	HI	Str, Grw, Fin	Ross/male	SBM	384
Hartinger et al. [22]	7	HI	Str, Grw	Ross/mixed	SBM	216
Elangovan et al. [23]	8	HI	Str	Cobb/mixed	SBM	90
Kim et al. [24]	9	HI oil	Str, Grw	Ross/male	SBM, CGM	300
Pietras et al. [25]	10	TM	Fin	Ross/male	SBM, LM	64
Dabbou et al. [26]	11	HI oil	Str, Grw	Ross/male	SBM, CGM	200
Mutisya et al. [27]	12	HI	Str, Fin	Cobb/mixed	FM	120
Elahi et al. [28]	13	TM	Str, Fin	Ross/male	SBM	700
Benzertiha et al. [29]	14	TM	Str, Grw	Ross/female	SBM, RS, FM	300
Benzertiha et al. [30]	15	TM oil	Str, Grw	Ross/female	SBM	48
Khan et al. [31]	16	TM	Str, Grw	Ross	SBM, CM, GM, SM	100
Brede et al. [32]	17	HI	Str, Grw	Ross/male	SBM	240
Onsongo et al. [33]	18	HI	Str, Fin	Cobb/male	SBM, FM	288
Biasato et al. [34]	19	TM	Str, Grw, Fin	Ross/male	SBM, CGM	160
Biasato et al. [35]	20	TM	Str, Grw, Fin	Ross/female	SBM, CGM	160
Bovera et al. [36]	21	TM	Fin	Shaver Brown/male	SBM	80
Józefiak et al. [37]	22	HI and TM	Str, Grw	Ross/female	SBM, RS, rye, FM	300
Schiavone et al. [38]	23	HI oil	Str, Grw, Fin	Ross/male	SBM, GLM	150
Schiavone et al. [39]	24	HI oil	Fin	Ross/male	SBM, GLM	120
Kareem et al. [40]	25	HI	Str, Grw, Fin	Cobb/female	SBM, FM	216

^1^ HI = *Hermetia illucens* and TM = *Tenebrio molitor* in meal form unless indicated as oil. ^2^ Feeding phases = Str: starter; Grw: grower; Fin: finisher. ^3^ Sex was informed whenever indicated in the manuscripts. ^4^ Protein sources in the control treatment; FM = fish meal; SBM = soybean meal; CGM = corn gluten meal; LM = lupine meal; RS = rapeseed meal; GM = guar meal; SM = sunflower meal; CM = cottonseed meal; GLM = gluten meal; ^5^ *n*: number of birds in the experiment and fed control diets and insect products.

**Table 2:** Use of *Hermetia illucens* (black soldier fly) meal or oil as ingredients for broilers and their impacts on health parameters.

Reference	Overall ^1^	Inclusion	Main Effects ^3^
Ipema et al. [41]	Replacement of 8% of the diet in DM with a different provision of HI (meal + oil, live scattered on litter, whole dried in the feeder and whole dried scattered on litter), and impacts on broiler health and behavior.	5.76%	No difference was found for feather corticosterone, IgG, and IgM against keyhole limpet hemocyanin in plasma. Footpad dermatitis and litter quality were affected by the treatments.
Bongiorno et al. [42]	HI live larvae for local poultry and the effects on blood parameters.	10% live larvae supplementation on the ADFI ^2^	HI reduced leukocytes levels and increased monocytes and cholesterol levels. Higher gamma glutamyl transferase in blood samples, indicating an improvement in liver health status.
Mat et al. [17]	Different replacement levels of fishmeal with defatted HI meal for broilers and the effects on blood parameters.	4%, 8%, and 12%	Corpuscular volume, monocytes, red blood cells, granulocytes, hematocrits, hemoglobin, corpuscular hemoglobin concentration, red cell distribution width, platelet volume, and lymphocytes were affected by HI meal.
Kierończyk et al. [43]	HI oil replacing soybean oil and the effects on gut microbiota and immune characteristics.	1.67% and 3.34%	100% replacement increased the total number of *Clostridium leptum* subgroups and *Enterobacteriaceae* and *Lactobacillus* groups in crop. No effects on jejunal microbiota. Reduced *C. perfringens* and all other microorganisms except *Bacteroides* with 50% HI replacement. Reduced cholesterol and ALT in plasma. No effects on plasma immunoglobulins and IL.
Hartinger et al. [20]	HI oil replacing 50% or 100% of soybean oil and HI meal replacing a 15% CP diet for broilers. Evaluated the effects on ileum histomorphology and caeca microbial metabolites.	5.2%, 4.64% and 4% of HI meal and/or 0.75%, 1.5%, 0.64% and 1.28% of HI oil	HI meal decreased the biogenic amines Agmatine, Spermidine, Spermine, and ammonia in caeca contents. Ethanolamine was higher with the HI oil. HI oil increased the concentration of Agmatine in colon contents. HI meal and 50 or 100% oil replacement increased jejunal villus area and width compared to control diets.
Kim et al. [19]	Microwave-dried HI meal replacing 25% and 50% of SBM.	Starter: 7.5% and 15%. Grower: 7% and 14%. Finisher: 6.5% and 13%	SCFAs in caeca were higher in HI treatments (except butyrate), especially with 50% replacement. Blood triglycerides, monocytes, and red blood cell distribution were higher in 50% replacement. Decreased low-density lipoprotein was obtained.
Zhang et al. [44]	Immune responses using HI larvae meal for broilers experimentally infected with IBV.	1%, 5%, and 10%	Reduced IBV symptoms with 10% inclusion. At the tissue level, reduced IBV infection and lesions were presented. Increased survival rate of chicks. Improved proliferation of CD8+ lymphocytes.
Hartinger et al. [22]	0, 15%, and 30% of CP from SBM replaced with HI meal.	0, 4%, 10%	No differences in intestine morphology parameters.
de Souza Vilela et al. [45]	Increasing levels of full-fat HI meal for broilers and the effects on immune responses.	Starter: 2.5%, 5%, 7.5%, and 10%. Grower/Finisher: 5%, 10%, 15%, and 20%	Decreased blood lymphocytes at 21 d and decreased cytotoxic T cell CD3+CD8+ in jejunum.
Kim et al. [46]	Replacement of 50% and 100% of soybean oil with HI oil and the effects on intestinal health and blood profile.	1.5% and 3%	Higher ileum villus height with 50% replacement. Higher butyrate levels in caeca content with HI oil. Lowered lipase levels in blood samples.
Biasato et al. [47]	Use of defatted HI meal in broilers and the impact on gut health.	5%, 10%, and 15%	HI did not affect the relative abundance of Firmicutes and Bacteroidetes and the Firmicutes/Bacteroidetes ratio, but 15% HI caused higher relative abundance of *Proteobacteria*. L*-Ruminococcus* (*Ruminococcus* from *Lachnospiraceae* family), *Faecalibacterium*, *Blautia*, *Clostridium*, *Bacteroides*, *Roseburia*, and *Helicobacter* genera. *Lactobacillus* and *Ruminococcus* improved in 10% HI diet. The mucin staining was not affected by HI.
Kierończyk et al. [6]	Replacement of 25%, 50%, 75% and 100% of soybean oil with HI oil and the effects on broiler gut histomorphology.	Starter: 0.83%, 1.67%, 2.34%, and 3.34%. Finisher: 1.23%, 2.46%, 3.44% and 4.92%	No effects on histomorphology of duodenum, jejunum, and ileum. The HI oil reduced the jejunum and ileum weights relative to BW.
Ipema et al. [48]	Use of live larvae and impacts on health and behavior of broilers.	5% and 10% of ADFI ^2^ in DM with live larvae	No effects on hock burns, lameness, cleanliness, thigh scratches, tibia length, tibia fluctuating asymmetry, and tibia breaking strength. Only reduced the tibia width with 10% replacement.
Lee et al. [49]	Immune activity of broilers experimentally infected with *Salmonella* Gallinarum fed with HI larvae.	1%, 2%, and 3%	Higher presence of CD3+CD4+ T lymphocytes in spleen. Amplified spleen lymphocyte proliferation, increased lysozyme activity in serum, and increased survival rate of chicks with 3% inclusion.
Dabbou et al. [50]	HI defatted meal and impacts on blood traits, gut morphology, and histological features.	5%, 10%, and 15%	No effects on hematological and serum parameters. Diet with 15% HI decreased villi height, crypt depth, and V:C ratio compared to the other diets.
Schiavone et al. [39]	Replacement of 50% and 100% of soybean oil with HI oil for broilers (finisher phase) and effects on blood and gut morphology parameters.	3.43% and 6.87%	No differences in blood and histomorphology parameters.
Schiavone et al. [38]	50% and 100% of soybean oil replaced with HI oil and the effects on blood parameters.	2.91% and 5.85%	No differences observed in blood analyses.

^1^ HI = *Hermetia illucens*; SBM = soybean meal; IBV = avian infectious bronchitis virus; CP = crude protein. ^2^ ADFI = average daily feed intake. ^3^ IgG = immunoglobulin G; IgM = immunoglobulin M; SCFAs = short-chain fatty acids; ALT = alanine aminotransferase; IL = interleukin; BW = body weight; V:C = villus/crypt ratio.

**Table 3:** Use of *Tenebrio molitor* (yellow mealworm) meal or oil as ingredients for broilers and their effects on health parameters.

Reference	Overall ^1^	Inclusion	Main Effects ^2^
Sedgh-Gooya et al. [18]	Effects on histomorphology of broilers fed full-fat TM meal.	2.5% and 5%	No effects on histomorphology.
Sedgh-Gooya et al. [51]	Inclusion of TM meal in broiler diets and effects on caeca microbiota and blood parameters.	2.5% and 5%	Decreased blood albumin/globulin ratio with TM diets. *Escherichia coli* reduced with 5% TM inclusion.
Benzertiha et al. [29]	Full-fat TM meal supplemented in broiler diets and impacts on bird immune system.	0.2% and 0.3%	TM meal diets had same IgY and IgM levels as diets with salinomycin, but had increased IL-2 and TNF-α with 0.3% TM inclusion.
Biasato et al. [52]	TM meal and the effects on broiler intestinal microbiota and mucin.	5%, 10%, and 15%	Decreased relative abundance of Firmicutes/Bacteroidetes ratio and the relative abundance of *Clostridium*, *Coprococcus*, L-*Ruminococcus*, and *Ruminococcus* with 15% TM. Higher mucin staining with 5% TM.
Elahi et al. [28]	Different levels of dried TM meal and usage of fresh TM meal.	2%, 4%, 8%, and 10.48% as fresh matter that correspond to 4% of dried TM inclusion	Linear increase in ALT in blood. Reduced total protein. Higher malondialdehyde and lower total antioxidant capacity. Higher uric acid in blood. Decreased levels of lysozyme with fresh TM.
Józefiak et al. [53]	Full-fat TM meal supplemented in broiler diets and effects on caeca microbiome.	0.2% and 0.3%	Phylum: Decreased relative number of Actinobacteria with 0.2% TM and Proteobacteria in both treatments. Increased Bacteroidetes with TM addition. Class: 0.3% TM decreased Clostridia and 0.2% TM increased Clostridia and decreased Actinobacteria. Order: 0.2% TM increased Clostridiales, but there was a decrease with 0.3% TM, and 0.2% TM decreased Lactobacilales. Family: 0.3% TM reduced *Ruminococcaceae*, *Enterobacteriaceae* and *Bifidobacteriaceae*. Genus: 0.3% TM stimulated growth of *Ruminococcus* and *Bifidobacterium*, and 0.2% TM decreased *Lactobacillus.*
Biasato et al. [54]	TM full-fat meal inclusion in broiler diets and effects on caeca microbiota and health.	5% and 15%	Higher inclusion of TM meal decreased relative abundance of Firmicutes phylum and Firmicutes/Bacteroidetes ratio. Higher inclusion of TM meal decreased villi mucin staining.
Benzertiha et al. [30]	Full-fat TM meal supplementation in diets and effects on enzyme activity and microbiota.	0.2% or 0.3%	TM meal at 0.3% decreased the caeca *Bacteroides– Prevotella* cluster. Also, 0.2 and 0.3% decreased *C. perfringens*. Salinomycin and TM treatments had the lowest extracellular β-glucuronidase and a higher α-glucosidase activity for caeca contents.
Benzertiha et al. [55]	TM oil and effects on pancreatic enzyme and blood parameters.	5%	TM oil reduced amylase activity and triglycerides in blood, and triglycerides and total cholesterol in liver.
Loponte et al. [56]	Total replacement of SBM with full-fat TM meal in broiler diets and the effects on caeca VFAs.	100% SBM replacement	Almost doubled Mmol/L in all VFAs, increased % of butyrate in total VFAs, and decreased acetate, propionate, and valerianate.
Biasato et al. [57]	TM meal and effects on intestinal microbiota, histomorphology, and mucin composition of free-range broilers.	7.5%	Increased *Sutterella*, *Ruminococcus*, *Oscillospira*, *Clostridium*, *Coprococcus*, and Firmicutes/Bacteroidetes ratio. Increased mucin staining intensity on ileum.
Biasato et al. [35]	TM meal and the effects on broiler health.	5%, 10%, and 15%	Increased levels of erythrocytes, linear decrease in albumin levels, and quadratic decrease in gamma glutamyl transferase in blood. No differences in histomorphology using TM meal.
Islam and Yang. [58]	TM probiotic supplementation for broilers experimentally challenged with *Salmonella enteritis* and *E. coli.*	0.4% of TM probiotic	IgG and IgA levels were higher with the probiotic. Lower mortality of birds and lower presence of *E. coli* and *Salmonella* spp. in caeca microbiota with the probiotic.

^1^ TM = *Tenebrio molitor*; SBM = soybean meal; VFAs = volatile fatty acids. ^2^ IgY = immunoglobulin Y; IgM = immunoglobulin M; IL = interleukin; TNF-α = tumor necrosis factor alpha; ALT = alanine aminotransferase; V:C = villus/crypt ratio; IgG = immunoglobulin G; IgA = immunoglobulin A.

**Table 4:** Use of *Hermetia illucens* and *Tenebrio molitor* as ingredients for broilers and their impacts on health parameters.

Reference	Overall ^1^	Inclusion	Main Effects
Bellezza Oddon et al. [59]	Live TM and HI larvae and impacts on health.	5% of ADFI ^2^ with larvae of TM or HI	Gut histomorphology index and histopathological alterations were not influenced by HI and TM larvae. No effects on hematological and serum parameters.
Colombino et al. [60]	Live TM and HI larvae for broilers and effects on mucin, immune response, and caeca microbiota.	5% of the expected ADFI	TM diets had lower interleukin-2 expression compared to HI diets. Mucin was not affected by live larvae. HI and TM influenced the relative abundance of the *Victivillaceae* family. *Saccharibacteria* and *Clostridium* increased HI, *Collinsella* was more abundant in TM treatment, and *Eubacterium* increased in both diets.
Józefiak et al. [37]	TM and HI full-fat meal supplementation and impact on microbiota.	0.2% supplemented	Crop digesta: HI decreased *C. leptum* and increased *C. coccoides–Eubacterium rectale* cluster. *Lactobacillus* spp. and *Enterococcus* increased in TM and HI groups, while TM reduced *Bacteroides*–*Prevotella*. Ileal digesta: both insects increased the number of C. *coccoides*–E. *rectale* and *Lactobacillus* spp. *Enterococcus* spp. counts decreased in HI and increased in TM groups. Caeca digesta: *Bacteroides*–*Prevotella*, *Streptococcus* spp./*Lactococcus* spp., *C. coccoides*–*E. rectale* cluster, and *Lactobacillus* spp./*Enterococcus* spp. count increased with HI.

^1^ HI = *Hermetia illucens*; TM = *Tenebrio molitor.* ^2^ ADFI = average daily feed intake.

**Table 5:** Variation between control diets and dietary inclusions of HI or TM meal or oil for broilers.

Variation, % ^1^	Meal		*p*-Value ^4^	Oil		*p*-Value
	HI ^2^	TM ^3^		HI	TM	
ADFI	−10.70	7.20	<0.001	0.61	−4.25	0.036
ADG	−7.92	4.05	0.002	−0.51	0.81	0.888
FCR	−1.49	2.46	0.241	0.41	−3.56	0.049

^1^ Variation in percentage between control diets (zero) and treatment diets with insect products in meal or oil forms for all feeding phases; ADFI = average daily feed intake; ADG = average daily gain; FCR = feed conversion ratio. ^2^ HI = *Hermetia illucens*. ^3^ TM = *Tenebrio molitor*. ^4^ Probability of treatment effects; differences were considered significant at *p* ≤ 0.05.

**Table 6:** Effects of insect inclusion on broiler performance.

Variation, % ^1^	Meal ^2^			*p*-Value ^4^	Oil ^3^			*p*-Value
	Control	Low	High		Control	Low	High	
ADFI	94.42 ^a^	95.98 ^a^	85.62 ^b^	<0.001	98.44	97.85	97.17	0.620
ADG	92.46 ^a^	94.70 ^a^	83.41 ^b^	<0.001	97.12	97.83	96.85	0.545
FCR	107.1	109.7	107.9	0.647	102.4	101.8	101.7	0.576

^a,b^ Differences were considered significant at *p* ≤ 0.05. ^1^ Variation in percentage of each performance parameter with the best performance result; ADFI = average daily feed intake; ADG = average daily gain; FCR = feed conversion ratio. ^2^ Low (≤100 g/kg) or high (>100 g/kg) inclusion of insect meal. ^3^ Low (≤25 g/kg) or high (>25 g/kg) inclusion of insect oil. ^4^ Probability of treatment effects.

## Data Availability

The data presented in this study are available upon request from the corresponding author.

## Supplementary Materials

The following supporting information can be downloaded at: https://www.mdpi.com/article/10.3390/vetsci10120702/s1, Figure S1. Nutritional composition of different *Hermetia illucens* meal. Figure S2. Nutritional composition of different *Tenebrio molitor* meal. Figure S3. Total amino acids (%) of *Hermetia illucens* larvae meal. Figure S4. Total amino acids (%) of *Tenebrio molitor* larvae meal. References [82,83] are cited in Supplementary Materials.
