# Supplementary material for: A Systematic Review and Metanalysis on the Use of Hermetia illucens and Tenebrio molitor in Diets for Poultry
Source: Vet Sci. 2023 Dec 12;10(12):702. doi: 10.3390/vetsci10120702 (PMC10747995; doi:10.3390/vetsci10120702)
Supplement: Supplementary file 1 [file vetsci-10-00702-s001.zip › vetsci-2647806-supplementary.pdf]

Supplementary materials

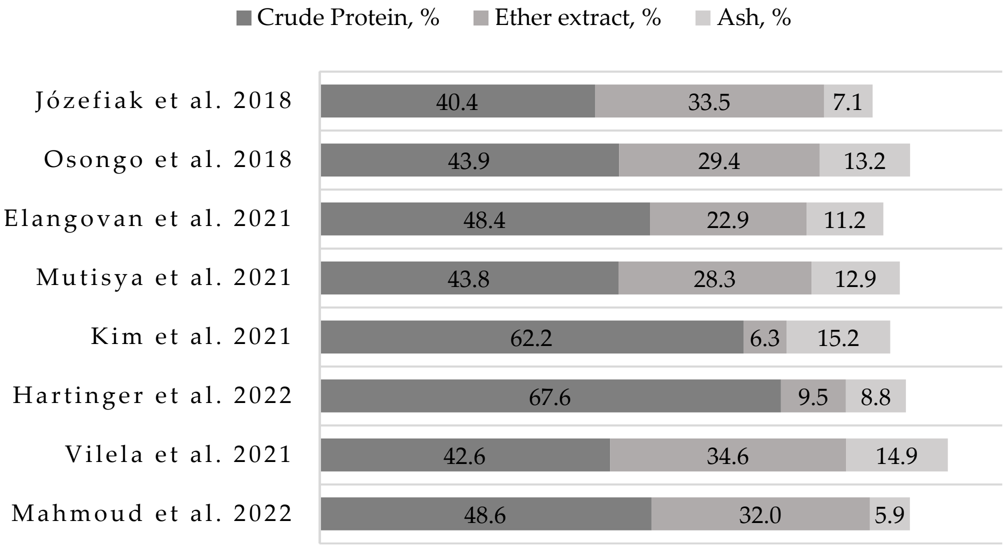

Publications: Kim et al., 2021 [46] defatted meal; Hartinger et al., 2022 [20]; Józefiak et al., 2018 [37]; Onsongo et al., 2018 [33]; Vilela et al., 2021 [45] Mahmoud et al., 2022 [82]; Mutisya et al., 2021 [27]; Elangovan et al., 2021 [23].

Figure S1. Nutritional composition of different *Hermetia illucens* meal.

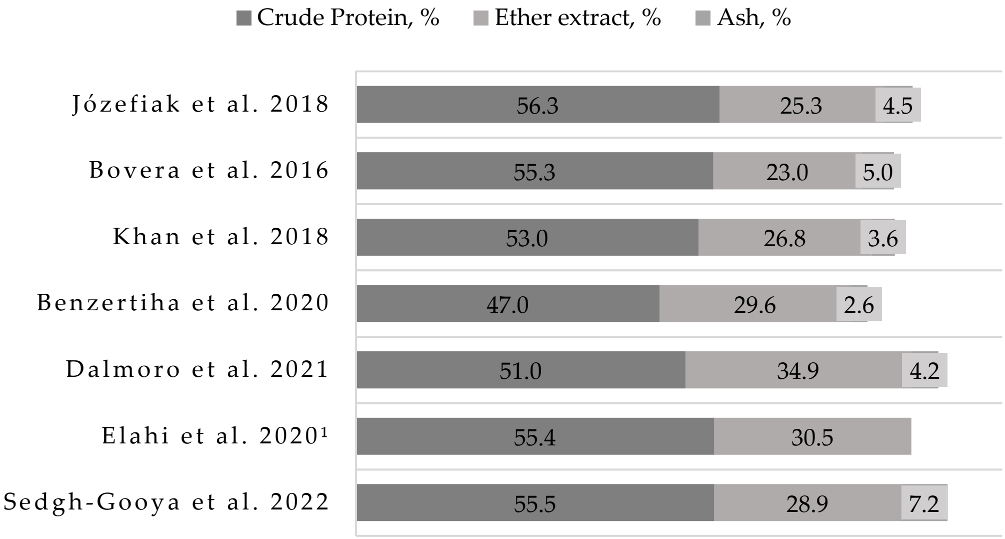

Publications: Elahi et al., 2020 [28][1] no data for ash composition; Józefiak et al., 2018 [37]; Bovera et al., 2016 [36]; Khan et al., 2018 [31]; Benzertiha et al., 2020 [29]; Dalmoro et al., 2021 [83]; Sedgh-Gooya et al., 2022 [18].

Figure S2. Nutritional composition of different *Tenebrio molitor* meal.

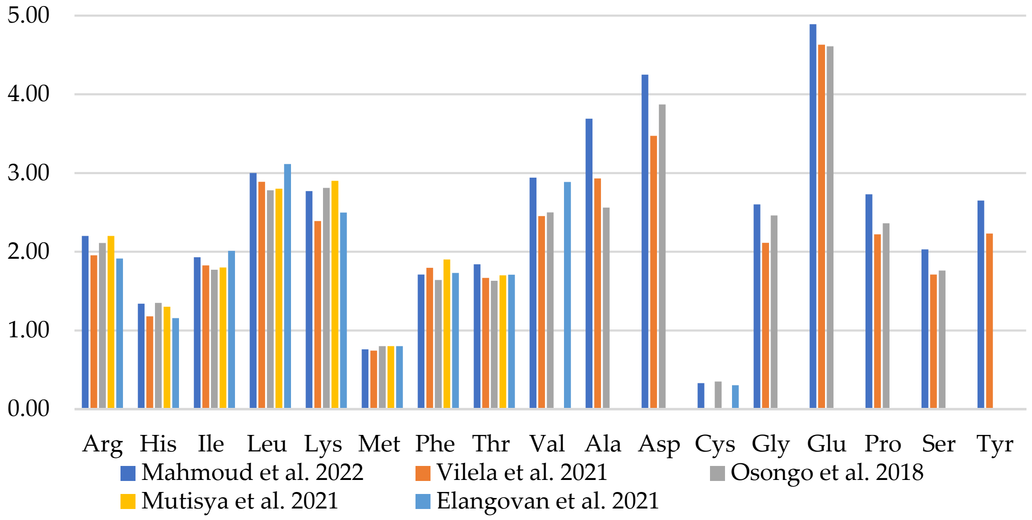

Publications: Vilela et al., 2021 [45], no data for cysteine; Osongo et al., 2018 [33] no data for tyrosine; Mutisya et al., 2021 [27] no data for valine, alanine, cysteine, glycine, glutamine, proline, serine, and tyrosine; Mahmoud et al., 2022 [82]; Elangovan et al., 2021 [23] no data for alanine.

Figure S3. Total amino acids (%) of *Hermetia illucens* larvae meal.

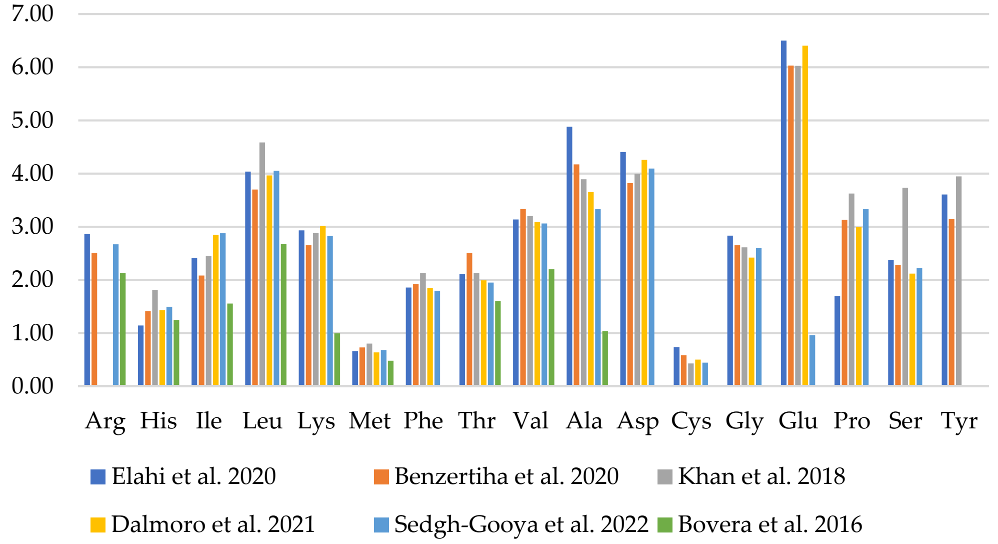

Publications: Khan et al., 2018 [31] no data for arginine; Dalmoro et al., 2021 [83] no data for arginine and tyrosine; Bovera et al., 2016 [36] no data for phenylalanine, aspartic acid, cysteine, glycine, glutamine, proline, serine, and tyrosine; Sedhgh-Gooya et al., 2021 [51] no data for tyrosine, aspartic acid, glycine, glutamine, proline, serine, and tyrosine; Elahi et al., 2020 [28]; Benzertiha et al., 2020 [29].

Figure S4. Total amino acids (%) of *Tenebrio molitor* larvae meal.

References

82. Mahmoud, A.E.; Morel, P.C.H.; Potter, M.A.; Ravindran, V. The Apparent Metabolisable Energy and Ileal Amino Digestibility of Black Soldier Fly (*Hermetia illucens*) Larvae Meal for Broiler Chickens. *Br. Poult. Sci.* **2023**, *64*, 377–383. https://doi.org/10.1080/00071668.2022.2161873.
83. Dalmoro, Y.K.; Adams, C.B.; Haetinger, V.S.; Bairros, L.; Yacoubi, N.; Stefanello, C. Energy Values of *Tenebrio molitor* Larvae Meal and Tilapia Byproduct Meal for Broiler Chickens Determined Using the Regression Method. *Anim. Feed Sci. Technol.* **2021**, *272*, 114784. https://doi.org/10.1016/j.anifeedsci.2020.114784.
